# Integrative single-cell and spatial transcriptomics analysis reveals MDK-NCL pathway’s role in shaping the immunosuppressive environment of lung adenocarcinoma

**DOI:** 10.3389/fimmu.2025.1546382

**Published:** 2025-05-06

**Authors:** Yu Fu, Song Li, Yikang Zhao, Xiran Zhang, Xiaolu Mao, Ran Xu

**Affiliations:** Department of Thoracic Surgery, Shengjing Hospital of China Medical University, Shenyang, China

**Keywords:** lung adenocarcinoma, tumor microenvironment, MDK-NCL, single-cell transcriptomics, spatial transcriptomics

## Abstract

**Objectives:**

The tumor microenvironment (TME) plays a critical role in the progression of lung adenocarcinoma (LUAD). This study aims to investigate the cellular composition of the TME in LUAD and assess the role of the MDK-NCL signaling pathway.

**Methods:**

We employed a multi-omics strategy to investigate LUAD, combining single-cell RNA sequencing (scRNA-seq), spatial transcriptomics (ST), and bulk RNA-seq datasets. Publicly available scRNA-seq data and ST data were utilized. scRNA-seq data underwent quality control, dimensionality reduction, and clustering to characterize cell populations and identify malignant epithelial subtypes using the Seurat and inferCNV packages. Spatial transcriptomics data facilitated the identification of distinct tumor niches, while immune infiltration and ligand-receptor interactions were analyzed using MCPcounter and Niches. Experimental validation was performed via real-time PCR and western blotting on paired LUAD and adjacent normal tissue samples.

**Results:**

scRNA-seq revealed the presence of multiple immune and stromal cell populations, with malignant epithelial cells being subdivided into six clusters. The MDK-NCL axis demonstrated high activity in malignant cells, showing strong interactions with immune and stromal components. Spatial transcriptomics revealed nine distinct tumor niches, with MDK-NCL signaling notably upregulated at the tumor-immune interface, highlighting its role in establishing an immunosuppressive microenvironment. In both the TCGA-LUAD cohort and in-house cohort, MDK and NCL were significantly upregulated at the mRNA and protein levels in tumor samples compared to normal tissues. High MDK-NCL expression in the TCGA-LUAD cohort correlated with increased TMB, MSI, and reduced immune cell infiltration. Elevated levels of immune checkpoint genes, including PD-1 and CTLA-4, in patients with high MDK-NCL expression suggested a potential resistance to immune checkpoint inhibitors. Moreover, patients with high MDK-NCL expression exhibited poorer survival outcomes, underscoring the pathway’s role in tumor progression and immune evasion.

**Conclusion:**

Our findings reveal that LUAD cells use the MDK-NCL signaling pathway to shape the TME, suppressing immune activity and promoting malignancy in epithelial cells. This study highlights the MDK-NCL axis as a potential therapeutic target for LUAD, particularly for patients with high MDK-NCL expression.

## Introduction

Lung adenocarcinoma (LUAD) is the most common subtype of non-small cell lung cancer (NSCLC), accounting for approximately 40% of lung cancer cases (1). While targeted therapies and immunotherapies have significantly improved the survival rates of some LUAD patients, the overall prognosis remains poor. This is primarily attributed to the tumor’s heterogeneity and the complexity of its tumor microenvironment (TME) (2). The TME, which consists of immune cells, stromal cells, extracellular matrix, and various signaling molecules, plays a critical role in tumor progression, immune evasion, and therapeutic resistance (3). Therefore, gaining a deeper understanding of the interactions between the tumor and its microenvironment is crucial for uncovering the mechanisms underlying cancer development and for the development of new therapeutic strategies.

Single-cell RNA sequencing (scRNA-seq) technology offers an unprecedented level of detail for deciphering the cellular heterogeneity and dynamic changes within tumors, enabling the identification of distinct cell types and their specific roles in the TME (4). In recent years, scRNA-seq has been widely employed in LUAD research, leading to the discovery of multiple heterogeneous cell subpopulations, including tumor cells, immune cells, and stromal cells, further elucidating mechanisms of immune evasion and the interactions between tumors and their microenvironment (5, 6). Moreover, the application of spatial transcriptomics has allowed for a more comprehensive understanding of the spatial distribution of these cell populations within tumors and their interactions, providing a more complete view of the TME (7, 8).

Among the many signaling pathways that influence the TME, the Midkine (MDK)-Nucleolin (NCL) axis has garnered significant attention in recent years. MDK, a pro-tumor growth factor, is highly expressed in various types of cancer and has been shown to promote cell proliferation, migration, and survival (9–11).In LUAD, MDK expression correlates with poor prognosis, yet its potential role in modulating immune suppression remains unclear (13). Unlike TGF-β-mediated immunosuppression, which primarily acts via Treg activation and myeloid suppression, MDK-NCL signaling may establish a distinct immunosuppressive niche by interacting with tumor-associated macrophages (TAMs) and fibroblasts. Research has demonstrated that the MDK-NCL axis facilitates the formation of an immunosuppressive microenvironment, thereby promoting immune evasion by tumor cells and contributing to tumor progression (12). Given these unique properties, investigating the MDK-NCL axis may reveal novel mechanisms of immune evasion in LUAD.

In this study, we utilized scRNA-seq and spatial transcriptomics to deeply analyze the TME in LUAD and further classify malignant cell populations. We identified that MDK-NCL signaling plays a critical role in the interactions between maliganant cells and immune cells, potentially driving immune evasion and reshaping the microenvironment. Through spatial transcriptomic data, we further revealed the differential spatial distribution of MDK-NCL signaling across various tumor niches. Moreover, by integrating bulk RNA-seq data from the TCGA-LUAD cohort, we investigated the relationship between MDK-NCL expression, immune cell infiltration, and clinical outcomes. This study provides new insights into the role of the MDK-NCL axis in LUAD, particularly regarding its involvement in microenvironmental remodeling and immune evasion. Our findings offer a theoretical foundation for considering MDK-NCL as a potential therapeutic target, with significant implications for enhancing the efficacy of immunotherapy in clinical settings.

## Results

### ScRNA-seq and cell type identification of LUAD

**Figure 1:**
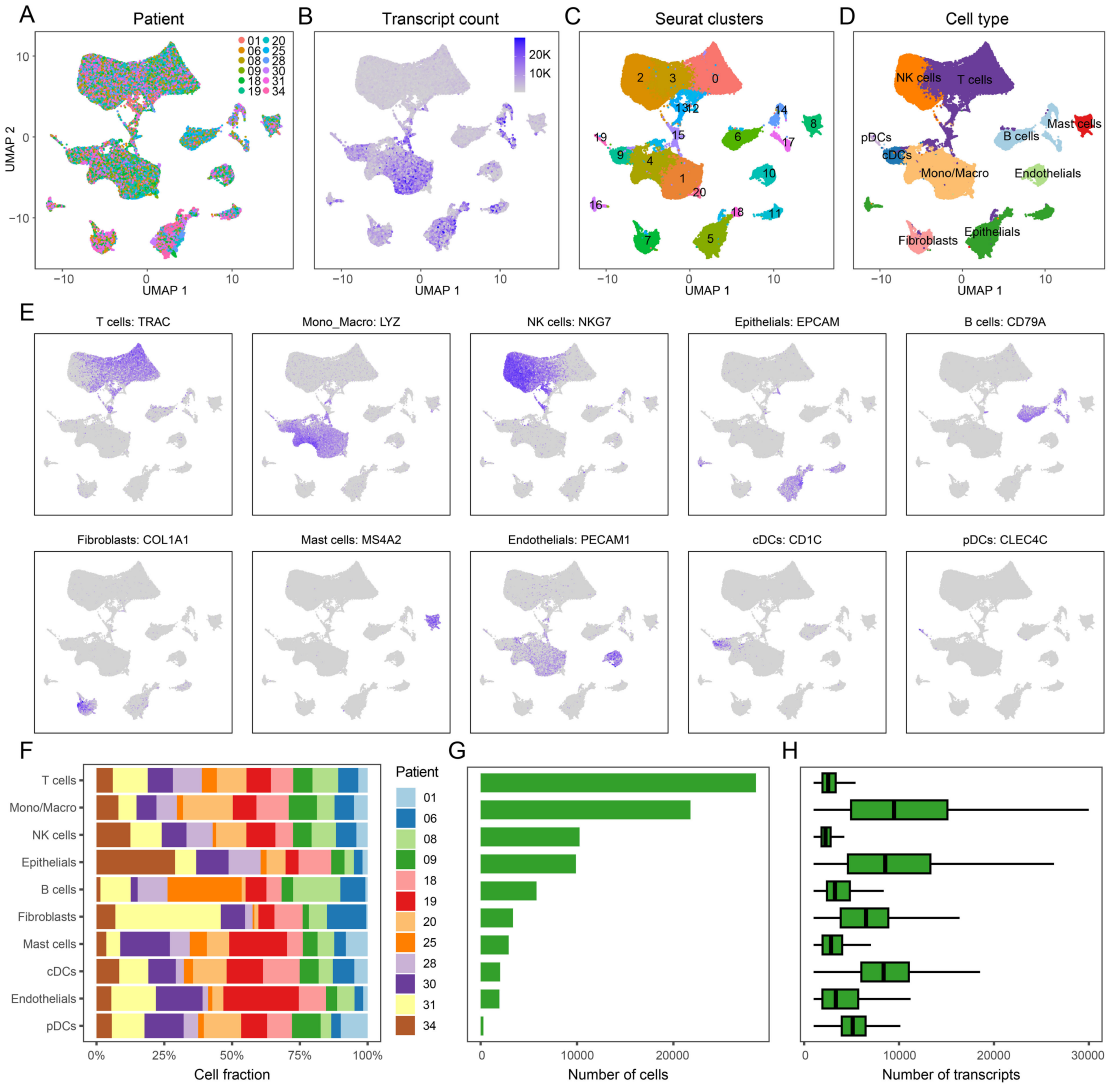
Annotation Results of scRNA-seq for LUAD. **(A)** Sample origin of the single-cell data, 12 samples were identified without batch effect. **(B)** Transcript counts in the single-cell dataset. **(C)** Clustering results of the single-cell data, totally 21 clusters were presented. **(D)** Cell type annotation based on marker gene expression, including T cells, monocyte-macrophages, NK cells, epithelial cells, B cells, fibroblasts, mast cells, endothelial cells, conventional dendritic cells (cDCs) and plasmacytoid dendritic cells (pDCs). **(E)** Expression profiles of representative markers for ten distinct cell types. **(F)** Proportion of each cell type across samples. **(G)** Total number of cells for each identified cell type. **(H)** Transcript counts per cell type, reflecting transcriptional activity at the single-cell level.

After correcting for batch effects, performing dimensionality reduction, and clustering, we analyzed several key aspects of the single-cell data (GSE131907). We visualized sample origins (**Figure 1A**), transcript counts (**Figure 1B**), cell clusters (**Figure 1C**), and cell type annotations (**Figure 1D**). Marker gene expression patterns, used to identify different cell types, are depicted in **Figure 1E**. Specifically, T cells were identified by TRAC, monocyte-macrophages by LYZ, NK cells by NKG7, epithelial cells by EPCAM, B cells by CD79A, fibroblasts by COL1A1, mast cells by MS4A2, endothelial cells by PECAM1, conventional dendritic cells (cDCs) by CD1C, and plasmacytoid dendritic cells (pDCs) by CLEC4C. The proportions of each cell type across samples are shown in **Figure 1F**, with the absolute numbers in **Figure 1G**, and transcript counts for each cell type detailed in **Figure 1H**. The same cell annotation procedure was also performed on the single-cell validation data GSE153935 (**Supplementary Figure S1A-E**).

### Malignant cell subpopulations in LUAD and their characteristics

**Figure 2:**
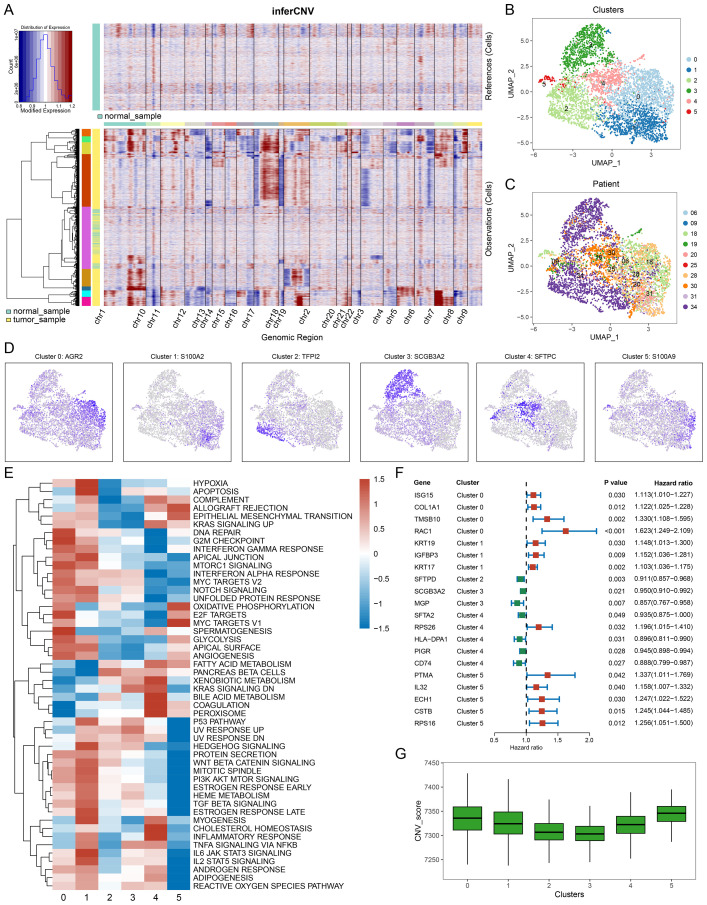
Identification of malignant cell subtypes. **(A)** inferCNV heatmap displaying copy number variations (CNVs) across cells, with normal samples in the upper panel and tumor samples in the lower panel. Red and blue indicate CNV gains and losses, respectively. **(B)** UMAP plot showing clustering of malignant cells, revealing distinct subpopulations. **(C)** UMAP plot indicating the patient origin of malignant cells, highlighting inter-sample heterogeneity. **(D)** Expression of representative marker genes for each malignant cluster: AGR2 (Cluster 0), S100A2 (Cluster 1), TPP2 (Cluster 2), SCGB3A2 (Cluster 3), SFTPC (Cluster 4), and S100A9 (Cluster 5). **(E)** Heatmap of hallmark pathway activities across clusters, with red indicating upregulation and blue indicating downregulation of pathways, such as hypoxia response and interferon signaling, cluster0 and cluster1 have more upregulated pathways. **(F)** Univariate Cox analysis of key marker genes, with hazard ratios, confidence intervals, and P-values showing their prognostic significance. Red indicates higher risk associations, while green indicates lower risk. **(G)** CNV scores of different malignant cell subtypes.

To infer malignancy within the epithelial cell populations, we applied inferCNV analysis in GSE131907 and GSE153935 (**Figure 2A** & **Supplementary Figure S1F**). In the GSE131907 dataset, malignant epithelial cells were subsequently extracted for further dimensionality reduction and clustering, revealing six distinct malignant cell subpopulations (clusters 0-5, **Figure 2B**). Sample distribution across these malignant clusters is illustrated in **Figure 2C**, highlighting both intra- and inter-sample heterogeneity within LUAD tumors.

Using the FindAllMarkers function, we identified cluster-specific markers for each malignant subpopulation (**Figure 2D**). Functional enrichment analysis via ssGSEA using hallmark gene sets revealed distinct biological pathways across clusters (**Figure 2E**). For example, clusters 0 and 1 were enriched in pathways related to metabolism and mitosis. Univariate Cox regression survival analysis, based on the top five markers per cluster, showed that markers from clusters 0, 1, and 5 were associated with higher risk, while clusters 2, 3, and 4 were more protective (**Figure 2F**). Meanwhile, clusters 0, 1, and 5 have higher CNV scores, suggesting greater genomic instability (**Figure 2G**). This suggests that clusters 0, 1, and 5 exhibit more aggressive, malignant phenotypes.

### The result of single-cell communication analysis

**Figure 3:**
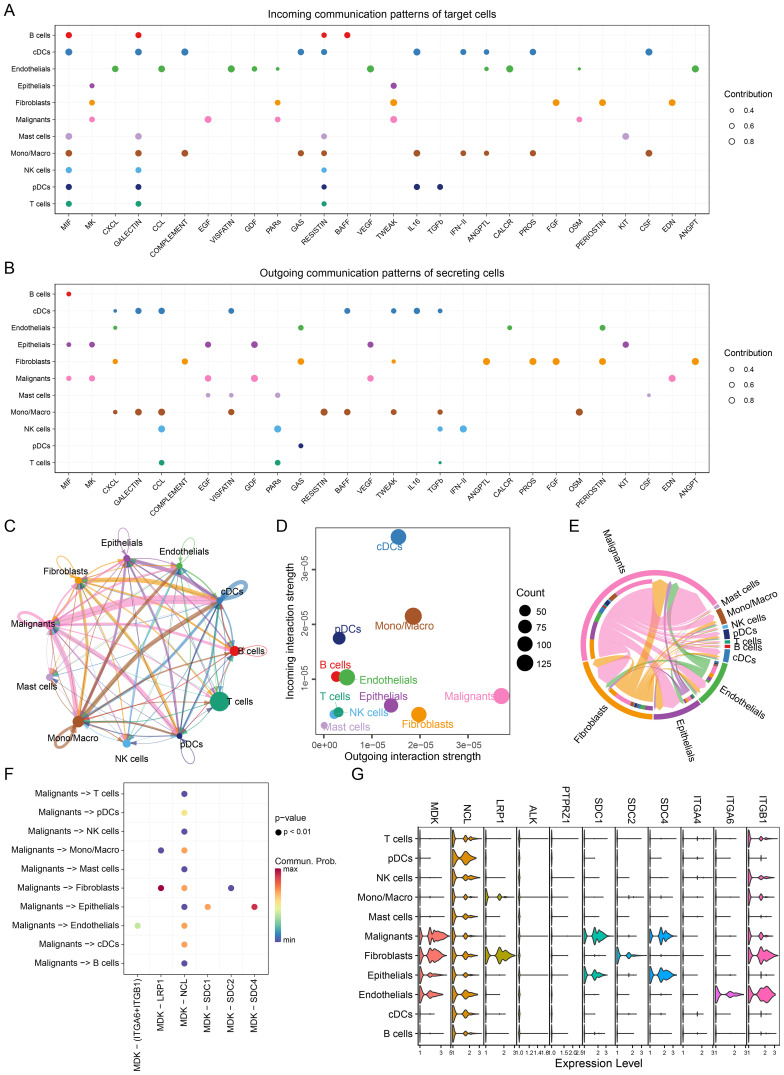
Single-cell communication networks. **(A)** Incoming communication patterns of target cells, showing pathways to which each cell type responds. **(B)** Outgoing communication patterns of secreting cells, illustrating the pathways through which cells send signals, MIF, MK and CXCL pathway exhibit high activity. **(C)** Network diagram showing the strength of intercellular communication, with connections between various cell types. **(D)** Scatter plot comparing outgoing and incoming communication strengths across cell populations, with bubble size indicating the number of interactions, malignant cells have higher strength of intercellular communication. **(E)** Chord diagram depicting communication via the MK pathway between different cell types. **(F)** Ligand-receptor interaction probabilities within the MK pathway between malignant and other cell types. Dot size represents significance (P-value), and color represents communication probability highlighting the MDK-NCL signaling pathway. **(G)** Violin plots of MK pathway gene expression levels across cell types, showing gene activity variations, MDK has advancer expression level in malignant cells.

Cell-to-cell communication was analyzed for both GSE131907 and GSE153935 using the CellChat package, which identified receptor-ligand signaling pathways received (**Figure 3A** & **Supplementary Figure S2A**) and emitted (**Figure 3B** & **Supplementary Figure S2B**) by different cell types. Notably, the MIF, MK, and CXCL signaling pathways were highly active. **Figure 3C** and **Supplementary Figure S2C** illustrates the overall communication strength between cell types, while **Figure 3D** and **Supplementary Figure S2D** shows the intensity of signals emitted and received by each cell type. Malignant cells exhibited the highest signal emission strength, underscoring their dominant role in influencing the TME.

Among receptor-ligand pairs, four of the top ten interactions belonged to the MK pathway, with the MDK-NCL interaction being the most significant (**Supplementary Figure S3** & **Supplementary Figure S4**). The strength of MK pathway communication across different cell types is presented in **Figure 3E** and **Supplementary Figure S2E**, with malignant cells being the primary senders and receivers of these signals. We further analyzed MDK-NCL interactions between malignant and immune/stromal cells, finding significant interaction strengths (**Figure 3F** & **Supplementary Figure S2F**). Malignant cells exhibited extensive interactions with all immune and stromal cell types through the MDK-NCL axis. Expression levels of genes involved in the MK pathway are shown in **Figure 3G**, with higher expression of MDK in malignant cells and broad expression of NCL across all cell types. These findings highlight the critical role of the MDK-NCL interaction in shaping the TME.

### Spatial transcriptomic niche communication analysis

**Figure 4:**
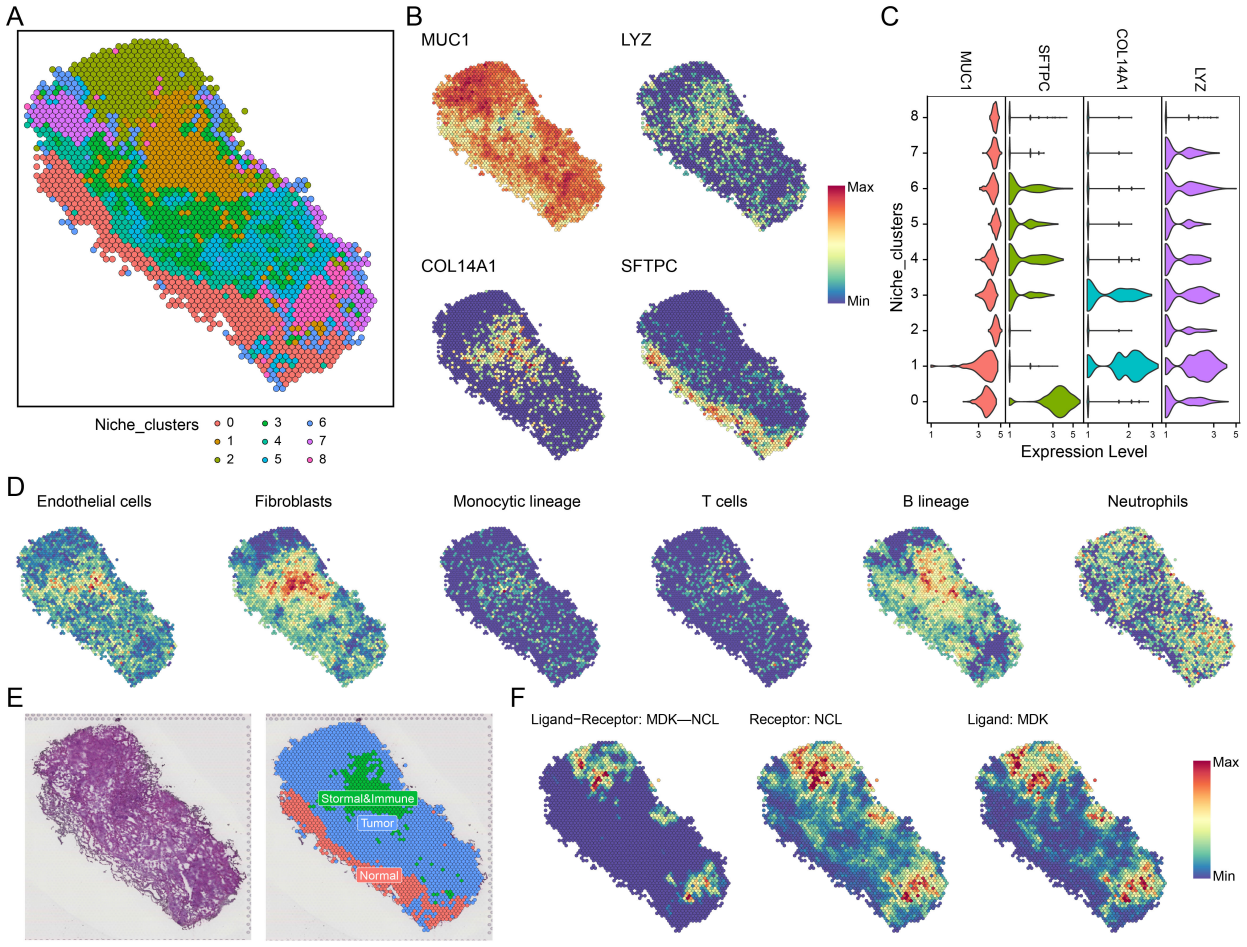
Spatial transcriptomics and MDK-NCL signal communication. **(A)** Niche clustering in spatial transcriptomics samples, identifying distinct ecological zones. **(B)** Spatial expression of representative markers in key regions: MUC1 (tumor region), LYZ (immune region), COL14A1 (stromal region), and SFTPC (normal region). **(C)** Violin plots displaying the expression of MUC1, LYZ, COL14A1, and SFTPC across different niches. **(D)** MCPcounter analysis showing the infiltration of six cell types (e.g., endothelial cells, fibroblasts, immune lineages) across spatial regions. **(E)** Spatial niche classification, distinguishing tumor, immune-stromal, and normal regions. **(F)** MDK-NCL ligand-receptor interaction analysis, spatially mapping MDK ligands, NCL receptors, and their binding regions.

Following dimensionality reduction and clustering, we identified nine distinct spatial niches (niche 0-8, **Figure 4A**). Based on the expression of key marker genes-MUC1 (tumor region), LYZ (immune region), COL14A1 (stromal region), and SFTPC (normal region)—we classified the niches into tumor, immune-stromal, and normal regions across all spatial transcriptomic samples (**Figures 4B, C**, **Supplementary Figure S5**). To validate our classification, we performed MCPcounter immune infiltration analysis (**Figure 4D**), identifying six distinct cell types-endothelial cells, fibroblasts, monocytes, T cells, B cells, and neutrophils-within the niches. The distribution of these cell types across the spatial niches is depicted in **Figure 4E**, showing a clear division into tumor, immune-stromal, and normal epithelial regions.

We then conducted spatial communication analysis to assess the spatial distribution of MDK-NCL receptor-ligand signaling across the niches (**Figure 4F**, **Supplementary Figure S5**). This included examining MDK-NCL ligand-receptor binding, NCL receptor levels, and MDK ligand expression. These spatial analyses revealed that the MDK-NCL axis plays a significant role in mediating communication between malignant cells and the surrounding immune and stromal cells, further contributing to the spatial organization of the TME.

### Pseudotime analysis of single cells

**Figure 5:**
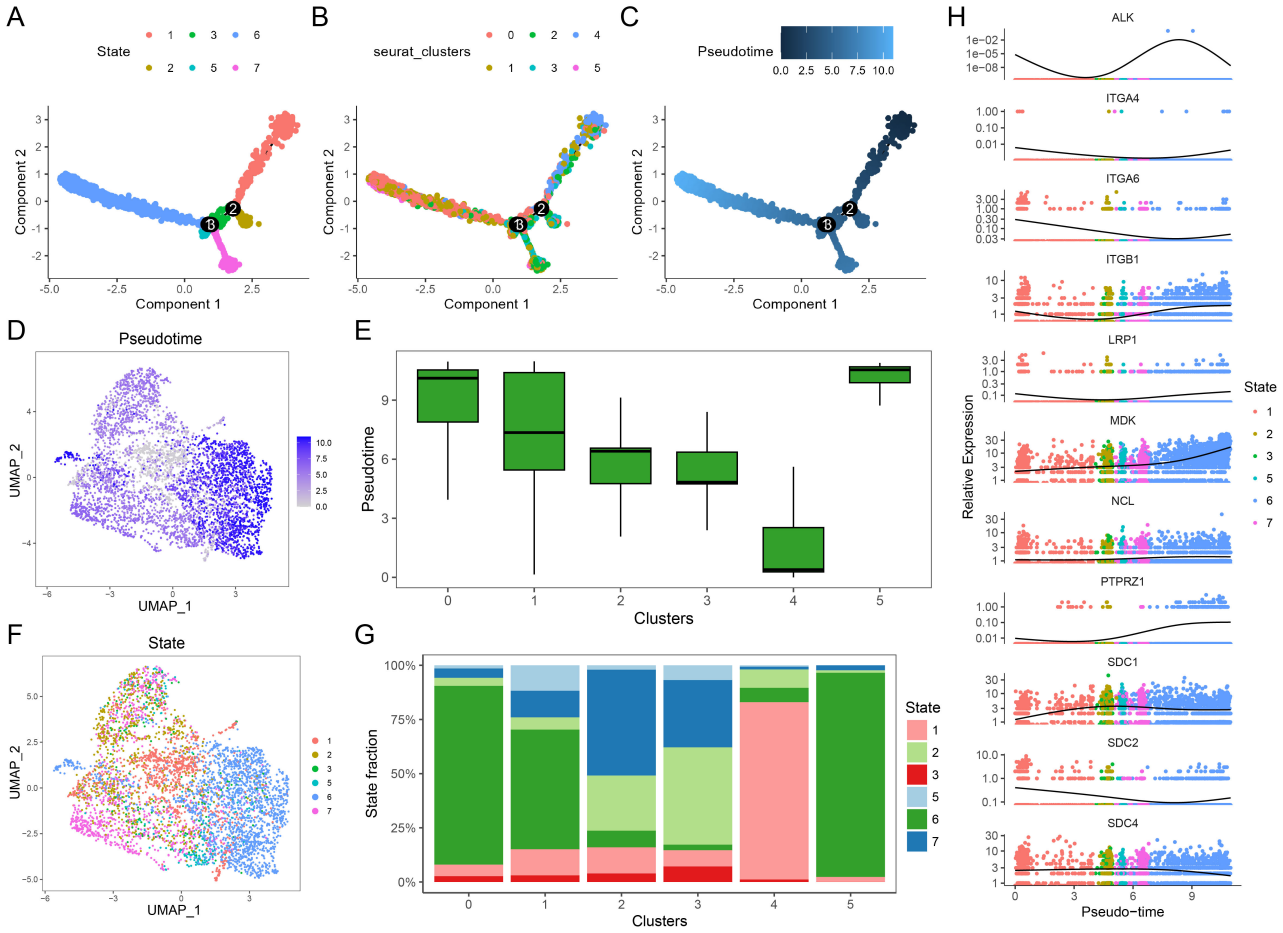
Single-cell pseudotime analysis. **(A)** Pseudotime trajectory analysis showing the 6 differentiation states of cells. **(B)** Subtype classification of malignant cells along the pseudotime trajectory. **(C)** Pseudotime scores mapped along the differentiation trajectory. **(D)** UMAP plot visualizing pseudotime scores across individual cells. **(E)** Box plots comparing pseudotime scores across different malignant cell clusters, cluster 0, 1, and 5 had higher pseudotime scores. **(F)** UMAP plot of differentiation states, with colors representing distinct states. **(G)** Stacked bar plots showing the proportion of differentiation states within each malignant cell cluster, cluster 0, 1, and 5 have larger proportion of state 6. **(H)** Expression dynamics of MK pathway genes (e.g., MDK, NCL, ITG genes) along the pseudotime trajectory, highlighting gene expression changes during differentiation, MDK and NCL express more in the later time.

To explore the developmental trajectory of malignant epithelial cells and the changes in the MK signaling pathway during tumor progression, we conducted a pseudotime analysis using spatial transcriptomics data from LUAD. **Figures 5A-C** illustrate the differentiation states, cell subtypes, and pseudotime scores obtained from the analysis. In **Figure 5D**, pseudotime scores are visualized using a UMAP dimensionality reduction plot, while a box plot (**Figure 5E**) compares the pseudotime scores of different malignant cell clusters, revealing that clusters 0, 1, and 5 have higher pseudotime scores. Additionally, the differentiation states of these clusters are shown in **Figure 5F**, and the proportion of cells in each state is presented in **Figure 5G**, with clusters 0, 1, and 5 primarily occupying differentiation state 6, which is associated with a more advanced pseudotime score. These findings indicate that clusters 0, 1, and 5, which are negatively correlated with prognosis, not only have higher pseudotime scores but also reside in more differentiated states, suggesting a higher level of tumor progression and malignancy. Finally, we analyzed the expression trends of MK pathway genes along the pseudotime trajectory (**Figure 5H**), which showed a gradual upregulation of MDK and NCL expression with increasing pseudotime scores.

### The impact of MDK-NCL on the LUAD immune microenvironment

**Figure 6:**
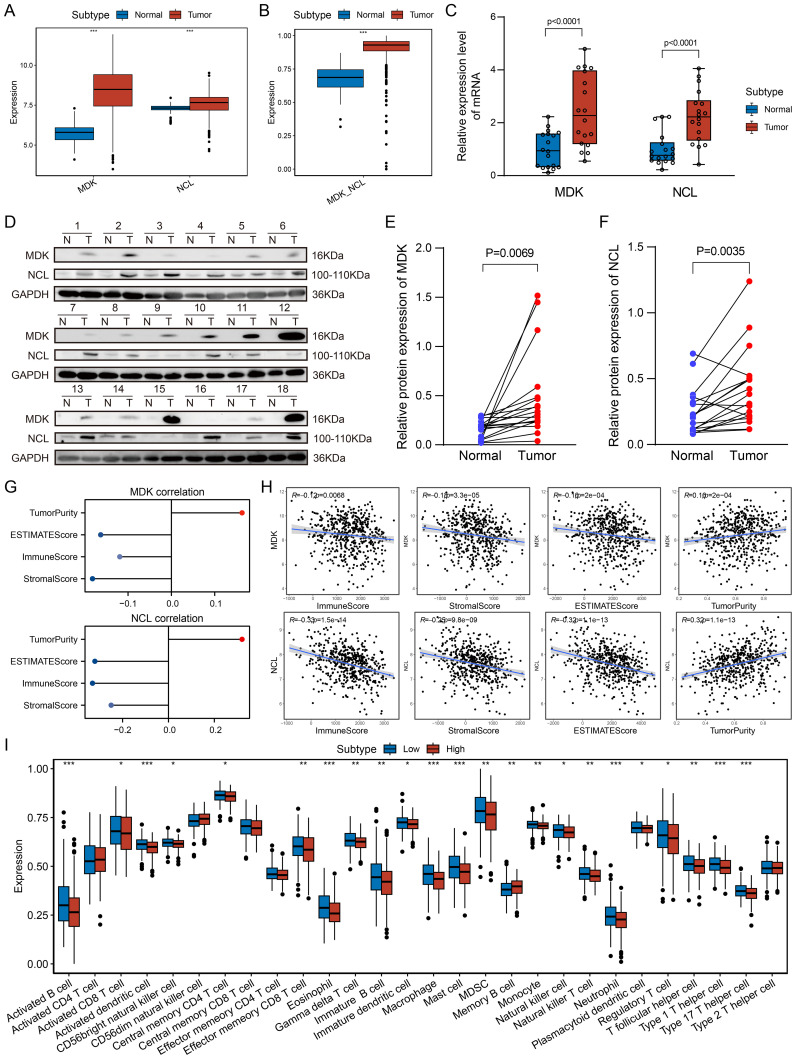
Association of MDK-NCL with the immune microenvironment. **(A)** Boxplot shows the expression levels of MDK and NCL genes in tumor and control groups, it exhibit higher activity in tumor group. **(B)** MDK-NCL enrichment scores in tumor and control groups. **(C)** Relative mRNA expression levels of MDK and NCL in tumor and control groups from in-house data. **(D)** Relative protein expression levels of MDK and NCL in tumor and control groups from in-house data. **(E)** Comparison of MDK protein expression levels between tumor and control groups. **(F)** Comparison of NCL protein expression levels between tumor and control groups. **(G)** Correlation of MDK and NCL expression with ImmuneScore, StromalScore, ESTIMATEScore, and TumorPurity. **(H)** Scatter plots depicting the relationship between MDK and NCL expression and immune-related scores (ImmuneScore, StromalScore, ESTIMATEScore) as well as TumorPurity. **(I)** Comparison of immune cell infiltration scores across high and low MDK-NCL expression groups for 28 immune cell types. *P < 0.05, **P < 0.01, ***P < 0.001.

Using single-cell and spatial transcriptomic analyses, MDK-NCL communication between tumor cells and other cells was identified as a critical mechanism in shaping the TME. Analysis of bulk transcriptomic data from the TCGA-LUAD cohort revealed that MDK and NCL expression levels were significantly higher in tumor samples compared to control samples (**Figure 6A**). Similarly, GSVA enrichment scores for the MDK-NCL pathway were also markedly elevated in tumor samples (**Figure 6B**). Three validation public cohorts were corresponding to the same results (**Supplementary Figure S6A-C**). Consistent with these findings, in our cohort, the relative mRNA expression levels of MDK and NCL were significantly higher in tumor tissues than in adjacent normal tissues (**Figure 6C**). Western blot analysis further confirmed that protein expression levels of MDK and NCL were significantly upregulated in tumor samples compared to controls (**Figures 6D-F**). To explore the impact of MDK and NCL on the immune microenvironment, we performed ESTIMATE analysis using the TCGA-LUAD dataset. The results demonstrated a negative correlation between MDK and NCL expression levels and immune-related scores, including the ImmuneScore, StromalScore, and ESTIMATEScore. Conversely, a positive correlation was observed between MDK and NCL expression and TumorPurity (**Figures 6G, H**). These findings suggest that MDK and NCL are associated with the development of an immunosuppressive TME. Further analysis divided tumor samples into high and low MDK-NCL expression groups based on the median enrichment score. Immune infiltration analysis revealed that immune cell scores for various cell types were significantly lower in the high MDK-NCL expression group compared to the low-expression group. Patients with high MDK-NCL expression groups exhibit increased infiltration of regulatory T cells (Tregs), myeloid-derived suppressor cells (MDSCs), and M2-like macrophages, which are known to promote immune evasion and tumor progression. Additionally, the MDK-NCL pathway suppresses cytotoxic immunity by reducing activated and effector memory CD8+ T cells while promoting an immunosuppressive microenvironment through increased Tregs and altering helper T cell differentiation, facilitating tumor immune evasion. This supports the conclusion that MDK-NCL activity suppresses immune cell infiltration and activity, contributing to immune evasion in LUAD.

### The association of MDK-NCL with immunotherapy

**Figure 7:**
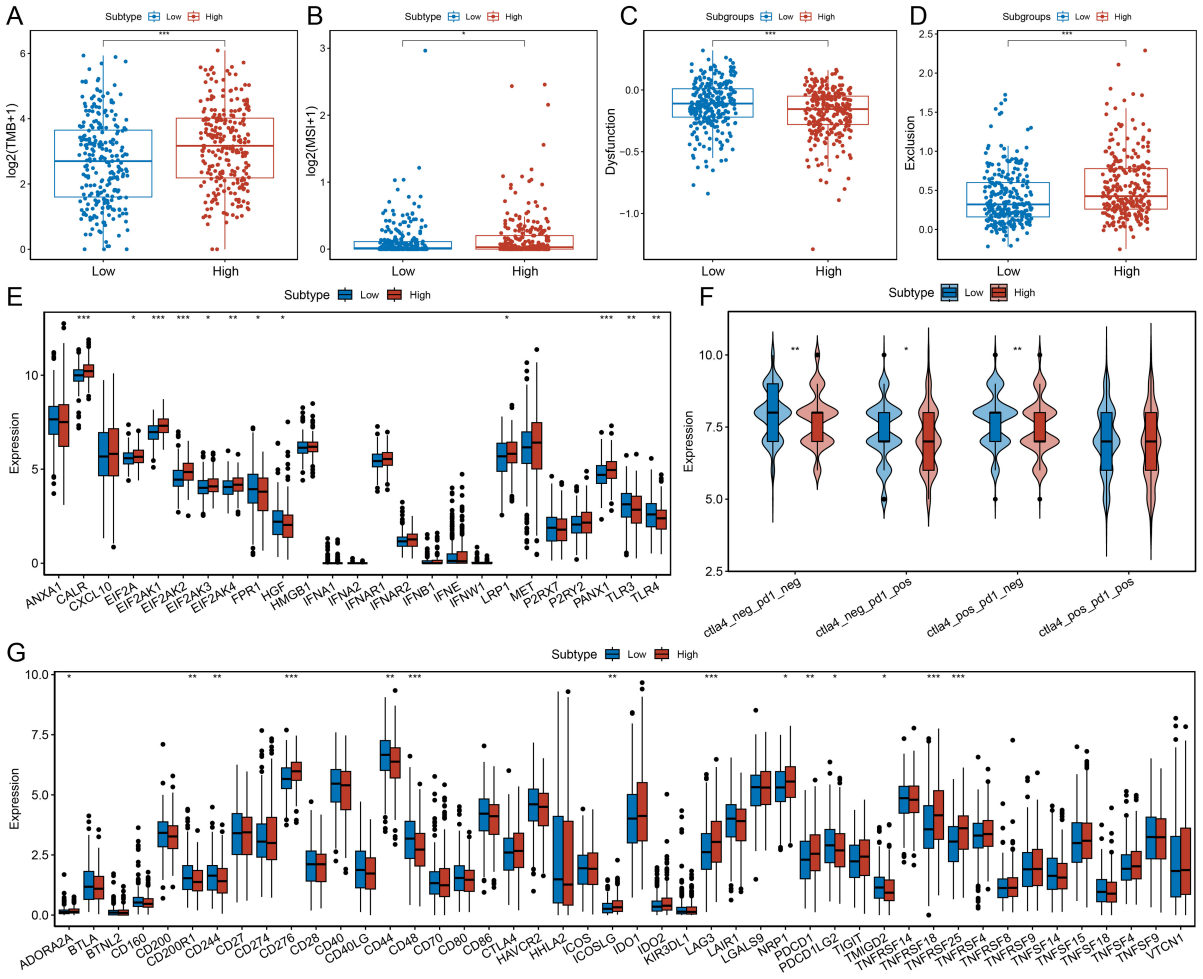
Association of MDK-NCL with immunotherapy response. **(A)** Comparison of tumor mutation burden (TMB) between high and low MDK-NCL expression groups. **(B)** Comparison of microsatellite instability (MSI) between high and low MDK-NCL groups. **(C)** Comparison of dysfunction scores between high and low MDK-NCL groups. **(D)** Comparison of exclusion scores between high and low MDK-NCL groups. **(E)** Expression of immunogenic cell death (ICD)-related genes in high and low MDK-NCL groups. **(F)** Expression levels of CTLA4 and PD1 in high and low MDK-NCL groups. **(G)** Comparison of immune checkpoint gene expression between high and low MDK-NCL expression groups. *P < 0.05, **P < 0.01, ***P < 0.001.

We observed that the high MDK-NCL expression group exhibited higher tumor mutation burden (TMB) (**Figure 7A**) and microsatellite instability (MSI) scores (**Figure 7B**), indicating increased genomic instability. TIDE analysis revealed that the high MDK-NCL group had lower Dysfunction scores (**Figure 7C**) and higher Exclusion scores (**Figure 7D**), suggesting that although T cell dysfunction was lower, there was a higher degree of T cell exhaustion. This supports that MDK-NCL may promote an immune-resistant TME through T cell exclusion rather than direct T cell exhaustion, a mechanism distinct from PD-1/PD-L1, which primarily induces T cell dysfunction at the tumor-immune interface. Additionally, we analyzed the expression patterns of immunogenic cell death (ICD)-related genes (**Figure 7E**), finding that the high MDK-NCL group had higher expression of several ICD genes, while toll-like receptors TLR3 and TLR4 showed lower expression. These findings suggest that MDK-NCL may contribute to immune evasion by promoting T cell exclusion and downregulating innate immune sensing, similar to TGF-β. Predictions from the TCIA database indicated that the high MDK-NCL group had fewer patients with dual-negative CTLA4 and PD1 status, as well as fewer patients with PD1 single positivity but more patients with CTLA4 single positivity (**Figure 7F**). This suggests that MDK-NCL may enhance CTLA-4 mediated immune suppression, potentially influencing the response to anti-CTLA-4 therapy. Similarly, most of these factors got the same trends in validation cohorts (**Supplementary Figure S6D-F**). Finally, we compared the expression profiles of immune checkpoint-related genes between the two groups (**Figure 7G**), revealing that the high MDK-NCL group had elevated expression of checkpoint genes such as LAG3 and PDCD1, suggesting that these patients may respond more favorably to immune checkpoint inhibitors. Overall, our findings suggest that high MDK-NCL expression may predict poor ICI response by fostering an immune-excluded tumor microenvironment. Despite high TMB/MSI, MDK-NCL-high tumors show low CD8+ T-cell infiltration and increased Tregs/MDSCs, potentially negating the benefits of increased neoantigens. This highlights MDK-NCL as a negative predictor of ICI response and a potential target to enhance ICI efficacy.

## Discussion

In this study, we systematically investigated the role of the MDK-NCL signaling axis in the TME of LUAD through the integration of scRNA-seq and spatial transcriptomics. Our findings shed light on the mechanisms by which the MDK-NCL pathway contributes to immune suppression and tumor immune evasion. offering novel insights into the potential of targeting this axis as a therapeutic strategy. This work deepens our understanding of TME remodeling in LUAD.

MDK, a pro-tumorigenic growth factor, is highly expressed in various cancers (10, 14). It exerts its oncogenic effects primarily by binding to its receptor, Nucleolin (NCL), through which it modulates various signaling pathways critical for the regulation of tumor progression and the maintenance of the TME (15, 16). Through scRNA-seq and spatial transcriptomics, we elucidated the role of MDK-NCL signaling in LUAD at the cellular level. Our results indicate that MDK-NCL plays a pivotal role in the interaction between malignant, immune, and stromal cells, particularly by fostering an immunosuppressive environment that supports tumor immune evasion. This mechanism is further supported by its observed spatial heterogeneity across distinct tumor regions. Spatial transcriptomics revealed that MDK-NCL signaling activity was markedly elevated at the tumor-immune interface, a region characterized by high cellular density and active immune-tumor interactions. This enrichment suggests that MDK-NCL may serve as a defensive mechanism for tumor cells at immune hotspots, preventing effective immune cell infiltration and cytotoxic activity. The differential expression across tumor niches underscores the biological importance of spatial heterogeneity in shaping TME architecture and influencing immune evasion strategies. For example, in low-immune regions, MDK-NCL may facilitate stromal remodeling, whereas in high-immune regions, it likely plays a more direct role in immune cell suppression. These observations align with previous studies emphasizing the role of spatial heterogeneity in defining TME functions (17). Understanding this spatial regulation not only highlights the complexity of MDK-NCL signaling but also opens avenues for spatially targeted therapeutic strategies, such as local delivery of inhibitors to high-activity regions within the TME.

Another important finding of this study is the potential role of the MDK-NCL axis in immunotherapy. By analyzing TMB and MSI data from the TCGA-LUAD dataset, we found that patients with high MDK-NCL expression tend to have higher TMB and MSI levels, indicating a potential association between MDK-NCL signaling and genomic instability, which may impact the response to immune checkpoint inhibitors (ICIs). While high TMB/MSI tumors are generally considered more immunogenic and respond better to ICIs, tumors with high MDK-NCL expression exhibit immune exclusion, despite their increased TMB/MSI levels. Specifically, we hypothesize that MDK-NCL blockade could enhance the effectiveness of anti-PD-1/PD-L1 and anti-CTLA-4 therapies by reversing T-cell exclusion and promoting immune cell infiltration. These findings highlight the potential dual role of MDK-NCL in influencing ICI responses. On one hand, increased TMB and MSI levels suggest heightened neoantigen production, which is typically associated with improved ICI efficacy. On the other hand, the observed high T-cell exclusion scores in patients with elevated MDK-NCL expression reflect an immunosuppressive phenotype, indicating that this axis might hinder the infiltration or activation of T cells despite a high mutational load. Additionally, elevated expression of immune checkpoint genes (e.g., PDCD1 and CTLA-4) in the high MDK-NCL group suggests that this pathway might promote immune evasion by enhancing the tumor’s dependence on checkpoint mechanisms. Therefore, targeting MDK-NCL signaling could potentially synergize with ICIs, alleviating immune suppression and restoring effective T-cell-mediated immunity. Moreover, the ability to stratify LUAD patients based on MDK-NCL expression into groups with distinct immune profiles and ICI responses could provide valuable insights for personalized therapy. Negative correlation between MDK-NCL expression and IFN-γ response genes, suggests that MDK-NCL may suppress IFN-γ-mediated antitumor immunity. Additionally, given the known role of TGF-β in promoting immune exclusion, MDK-NCL may interact with this pathway to reinforce immune suppression. For instance, patients with high MDK-NCL expression may benefit from combination therapies targeting both MDK-NCL signaling and immune checkpoints, improving response rates and reducing resistance to treatment. Future studies should focus on preclinical models to validate this hypothesis and assess the feasibility of such combination strategies in LUAD.

Preclinical studies have demonstrated the efficacy of MDK and NCL inhibitors in cancers such as glioblastoma (18) and pancreatic cancer (19), where MDK signaling is implicated in tumor progression and immune suppression. However, their clinical efficacy in LUAD remains unexplored. Our study highlights the critical role of the MDK-NCL axis in LUAD immune evasion and tumor progression, providing a theoretical basis for targeting this pathway as a novel therapeutic strategy. Our findings indicated that high MDK-NCL expression correlates with reduced infiltration of antigen-presenting cells (APCs), such as dendritic cells and MHC class I/II expression levels. This suggests that MDK-NCL signaling may downregulate antigen presentation, reducing tumor immunogenicity. However, further functional studies are required to confirm this hypothesis. The high expression of MDK-NCL signaling in LUAD patients is associated with an unfavorable immune microenvironment and increased immune exclusion, suggesting that targeting this axis may enhance the efficacy of existing immunotherapies. Unlike PD-1/PD-L1, which primarily induces T-cell exhaustion, our data suggest that MDK-NCL drives immune suppression through T-cell exclusion and stromal remodeling. Additionally, MDK-NCL-high tumors show increased infiltration of regulatory T cells (Tregs) and myeloid-derived suppressor cells (MDSCs), highlighting a distinct mechanism of immune evasion. Developing MDK-NCL pathway inhibitors holds promise as a novel treatment option for refractory LUAD, particularly for patients unresponsive to conventional immunotherapy.

Despite these significant findings, our study has several limitations. Although we integrated multi-omics data to explore the role of MDK-NCL signaling in LUAD and conducted preliminary experimental validation, further mechanistic studies are required, particularly in larger clinical cohorts. Additionally, our analysis relies heavily on publicly available datasets, which lack extensive clinical sample support. Prospective clinical studies are needed to validate our conclusions. Moreover, the development of MDK-NCL pathway inhibitors should be prioritized and evaluated in clinical trials. Finally, integrating additional multi-omics approaches, such as proteomics and metabolomics, could provide a more comprehensive understanding of the complex regulatory roles of MDK-NCL signaling in tumor progression and immune evasion.

## Methods

### Data acquisition

We downloaded spatial transcriptomics data from a LUAD patient sample using the 10x Visium technology from the BioStudies database (20) (https://www.ebi.ac.uk/biostudies/) (Accession number: E-MTAB-13530, This dataset includes a total of 40 lung tissue or NSCLC samples. For our analysis, we selected 8 tumor samples from this cohort.

From the Gene Expression Omnibus (GEO) database (21) (https://www.ncbi.nlm.nih.gov/geo/), we obtained the GSE131907 dataset (22) and GSE153935 dataset (23), which includes scRNA-seq data generated using the 10x Genomics platform and Drop-seq platform. GSE131907 dataset comprises 22 single-cell samples, including 11 primary tumor samples and 11 normal lung tissue samples, and was used for experimental analysis in this study. GSE153935 dataset comprises 18 single-cell samples, including 12 primary tumor samples and 6 normal lung tissue samples, and was used for validation analysis in this study.

We also retrieved bulk gene expression data (TPM) and clinical information such as patient gender, age, stage, grade, and survival outcomes from The Cancer Genome Atlas (TCGA) database (https://portal.gdc.cancer.gov/). Additionally, tumor mutation burden (TMB) and microsatellite instability (MSI) data for LUAD patients were obtained from cBioPortal (24) (https://www.cbioportal.org/). Meanwhile, GSE11969 (25) (including 94 LUAD and 5 normal samples), GSE43458 (26) (including 80 LUAD and 30 normal samples), GSE116959 (27) (including 57 LUAD and 11 normal samples) were obtained from GEO database as well for validation.

### Single-cell RNA-seq data processing for LUAD

We utilized the Seurat package (version 4.3.0) (4) to process and analyze the scRNA-seq data. Quality control was performed by filtering out cells with fewer than 200 or more than 8,000 genes, those with fewer than 50,000 transcripts, cells with greater than 20% ribosomal gene content, and cells with more than 3% hemoglobin gene expression.

Next, SCTransform (28) was applied for normalization and variance stabilization, followed by the Harmony algorithm (29) correct batch effects. Principal component analysis (PCA) was performed, and the first 30 principal components were used for clustering with the Louvain algorithm (resolution = 0.5) UMAP embedding was generated using default parameters (n.neighbors = 30) for visualization. Using characteristic gene markers, we classified the single-cell populations into T cells (CD3D, CD3E, TRAC), monocyte-macrophages (LYZ, CSF3R), NK cells (NKG7), epithelial cells (EPCAM), B cells (CD79A, MS4A1), fibroblasts (COL1A1, FN1), mast cells (MS4A2, TPSB2), endothelial cells (VWF, PECAM1), cDC cells (CD1C), and pDC cells (CLEC4C). Finally, we visualized the clinical information, clustering results, marker gene expression, and cell annotations using UMAP plot to display the reduced dimensions of the single-cell data.

### Identification of benign and malignant epithelial cells and subtyping of malignant epithelial cells

To distinguish malignant from benign epithelial cells, we applied inferCNV analysis (https://github.com/broadinstitute/inferCNV). We randomly selected 1,000 normal epithelial cells from control samples and inserted them into the tumor epithelial cell dataset. The remaining normal epithelial cells served as the reference. CNVs were inferred based on expression intensity across genomic regions, using denoise=TRUE and default settings. Cells displaying significant CNV patterns distinct from normal epithelial cells were classified as malignant, while those resembling reference cells were categorized as benign. The CNV scores of epithelial cells were also utilized to assist in distinguishing between benign and malignant epithelial cells.

After isolating all malignant cells, we performed further clustering to categorize them into distinct malignant cell clusters. Using the Seurat package’s “FindAllMarkers” function, we identified highly expressed marker genes for each cluster (log2FoldChange > 1, p value < 0.05). Subsequently, with hallmark gene sets from the MsigDB database (30), we applied single-sample gene set enrichment analysis (ssGSEA) via the GSVA package (31) to explore the biological functional characteristics of the malignant cell clusters. Additionally, univariate Cox regression analysis was performed to assess the prognostic significance of marker genes in each malignant cell cluster.

### Cell-cell communication analysis

To explore intercellular communication within the tumor microenvironment, we used the CellChat package (32). Receptor-ligand interactions were inferred using the computeCommunProb() function, with a minimum interaction probability threshold of 0.05 to filter out weak interactions. Pathway activity scores were generated using computeCommunProbPathway(), and the rankNet() function was applied to identify the most active signaling pathways. Significant interactions were visualized using netVisual_circle() and netVisual_aggregate(), highlighting key intercellular communication networks. The MDK-NCL signaling pathway emerged as a central interaction hub, particularly enriched in malignant epithelial and stromal cells, and was selected for further spatial and functional analysis.

### Processing of spatial transcriptomics data for LUAD

The spatial transcriptomics data were generated using the 10x Genomics Visium platform and processed using the Seurat package (4). Quality control was performed by removing spots with fewer than 500 detected genes or over 10% mitochondrial gene expression. Normalization and variance stabilization were conducted using SCTransform, followed by PCA for dimensionality reduction. The top 30 principal components were used for Louvain clustering (resolution = 0.5). After dimensionality reduction and clustering, we identified nine distinct spatial niches. Based on the expression of MUC1 (tumor region), LYZ (immune region), COL14A1 (stromal region), and SFTPC (normal region), we classified the niches into tumor, immune-stromal, and normal regions.

We then applied MCPcounter analysis (33) to assess the infiltration levels of various cell types (including T cells, B cells, neutrophils, monocytes, fibroblasts, and endothelial cells) in each spot of the spatial transcriptomics data. This allowed us to map the spatial distribution of immune infiltration and compare it with the defined niche regions.

Lastly, using the niches R package (34), we conducted spatial ligand-receptor interaction analysis, which integrates gene expression with spatial proximity. Interaction scores were computed for each ligand-receptor pair between neighboring spots, and only statistically significant pairs (adjusted p < 0.05) were retained for downstream analysis. Compared to single-cell analysis, spatial transcriptomics data incorporates spatial localization, providing more biologically accurate ligand-receptor interactions.

### Pseudotime analysis

Monocle (35) was used to construct pseudotime trajectories. The “orderCells” function assigned pseudotime values to each cell, and branching events were analyzed to assess transitions between malignant cell states. The MK signaling pathway activity was overlaid on the trajectory to observe its temporal dynamics.

### Immune-related analysis of TCGA-LUAD

Using the ESTIMATE package (36), we performed ESTIMATE analysis to assess the overall tumor immune microenvironment in each LUAD sample. This was achieved by calculating tumor purity, immune score, and stromal score. Additionally, the infiltration levels of 28 different immune cell types in each sample were evaluated using ssGSEA, and detail of 28 immune signature genes were shown in **Supplementary Table S1**.

### Therapy-related analysis of TCGA-LUAD

TMB and MSI are critical factors that influence the interaction between immune cells and tumor cells during immunotherapy. Numerous studies have demonstrated their role in predicting responses to immunotherapy. In this study, we explored the relationship between the MDK-NCL pathway and immunotherapy by comparing TMB and MSI between groups. We also conducted TIDE analysis (Tumor Immune Dysfunction and Exclusion) (37), a widely used method to assess the functional state of T cells in transcriptomic samples, and obtained two key metrics, Dysfunction and Exclusion, which reflect T cell functionality.

In addition, immunogenic cell death (ICD) is another key factor influencing immunotherapy efficacy. We compared the expression patterns of ICD-related genes between groups in TCGA and validation datasets. From the TCIA database (https://www.tcia.at/home), we retrieved predictions of CTLA4 and PDCD1 expression levels in TCGA-LUAD patients and conducted comparisons between the two groups. Lastly, we examined the differential expression profiles of immune checkpoint-related genes, which are closely associated with the response to immune checkpoint inhibitors, between the two groups.

### Sample collection

A total of 18 paired LUAD (lung adenocarcinoma) tissues and corresponding adjacent normal tissues were collected from patients undergoing surgical resection at Department of Thoracic Surgery, Shengjing Hospital of China Medical University. All patients included in the study had not received neoadjuvant therapy prior to surgery. The study was approved by the Ethics Committee of Shengjing Hospital, China Medical University (Approval No. 2024PS1727K).

### Real-time quantitative PCR

Total RNA was extracted from tissues using the Trizol reagent (R401-01, Vazyme, Nanjing, China) following the manufacturer’s protocol. Complementary DNA (cDNA) was synthesized from the extracted RNA using the reverse transcription kit (RR047A, TAKARA, Japan) according to the kit instructions. The relative expression levels of the target genes were determined using β-actin as the internal reference gene. Primer sequences for all genes are listed in **Supplementary Table S2**. All target gene expression analyses were performed in triplicate to ensure reproducibility.

### Western blot

LUAD and control tissue samples were homogenized using ultrasonic disruption and lysed for 30 minutes in RIPA lysis buffer (BL504A, Biosharp, China) containing PMSF (1:100, BL507A, Biosharp, China) and a protease inhibitor cocktail (1:50, P1082, Beyotime, China). Lysates were centrifuged at 12,000 rpm for 20 minutes, and protein concentrations were determined using the BCA protein assay kit (PC0020, Solarbio, China). Proteins were separated by SDS-PAGE (10% gel for MDK and 6% gel for NCL) and transferred onto PVDF membranes (IPVH00010, Millipore, USA). Membranes were blocked with 5% non-fat milk at room temperature for 2 hours and incubated overnight at 4°C with primary antibodies. Afterward, membranes were incubated with secondary antibodies for 2 hours at room temperature. Protein bands were visualized using enhanced chemiluminescence (ECL) reagent (BMU102, Abbkine, USA). Primary antibodies included MDK (1:1000, BM4392, BOSTER, Wuhan, China), NCL (1:1000, A00228-1, BOSTER, Wuhan, China), and GAPDH (1:1000, Sigma, USA), which was used as an internal control. The secondary antibody used was BA1039 (BOSTER, Wuhan, China). All protein bands were quantified using ImageJ software (Rawak Software Inc., Stuttgart, Germany).

### Statistical analysis

All data processing and statistical analyses were performed using R software (version 4.1.1). The Mann-Whitney U test (also known as the Wilcoxon rank-sum test) was used to evaluate differences between non-normally distributed variables. Spearman correlation analysis was employed to calculate correlation coefficients between non-normally distributed data. A p-value of less than 0.05 was considered statistically significant.

## Data Availability

The original contributions presented in the study are included in the article/**Supplementary Material**. Further inquiries can be directed to the corresponding author.

## Supplementary material

Supplementary Figure 1 Annotation results of scRNA-seq dataset GSE153935 for LUAD. **(A)** Sample origin of the GSE153935. **(B)** Transcript counts in the GSE153935. **(C)** Clustering results of the GSE153935. **(D)** Cell type annotation based on marker gene expression. **(E)** Expression profiles of representative markers for ten distinct cell types. **(F)** InferCNV heatmap displaying CNVs across cells, with normal samples in the upper panel and tumor samples in the lower panel. Red and blue indicate CNV gains and losses, respectively.

Supplementary Figure 2 Single-Cell communication networks for validation dataset. **(A)** Incoming communication patterns of target cells, showing pathways to which each cell type responds. **(B)** Outgoing communication patterns of secreting cells, illustrating the pathways through which cells send signals. **(C)** Network diagram showing the strength of intercellular communication, with connections between various cell types. **(D)** Scatter plot comparing outgoing and incoming communication strengths across cell populations, with bubble size indicating the number of interactions. **(E)** Chord diagram depicting communication via the MK pathway between different cell types. **(F)** Ligand-receptor interaction probabilities within the MK pathway between malignant and other cell types. Dot size represents significance, and color represents communication probability. **(G)** Violin plots of MK pathway gene expression levels across cell types, showing gene activity variations.

Supplementary Figure 3 Contribution of each ligand-receptor pair for GSE131907 dataset.

Supplementary Figure 4 Contribution of each ligand-receptor pair for GSE153935 dataset.

Supplementary Figure 5 The spatial distribution of MDK-NCL receptor-ligand signaling across the niches.

Supplementary Figure 6 MDK-NCL ligand-receptor interaction analysis, spatially mapping MDK ligands, NCL receptors, and their binding regions in validation data.
