# Supplementary figures and images for: Integrative single-cell and spatial transcriptomics analysis reveals MDK-NCL pathway’s role in shaping the immunosuppressive environment of lung adenocarcinoma
Source: Front Immunol. 2025 May 6;16:1546382. doi: 10.3389/fimmu.2025.1546382 (PMC12089103; doi:10.3389/fimmu.2025.1546382)

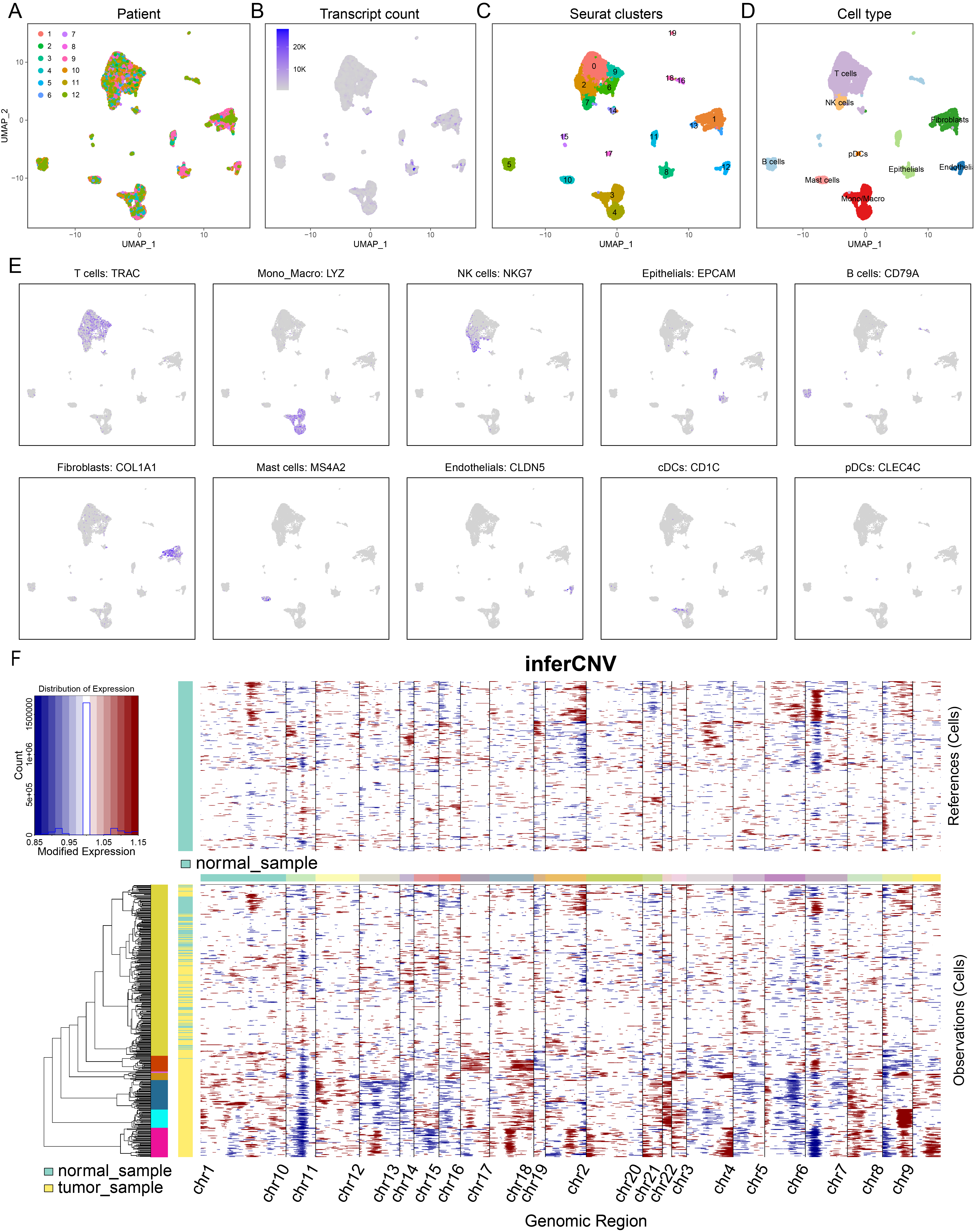

Supplement: Supplementary Figure 1 — Annotation results of scRNA-seq dataset GSE153935 for LUAD. (A) Sample origin of the GSE153935. (B) Transcript counts in the GSE153935. (C) Clustering results of the GSE153935. (D) Cell type annotation based on marker gene expression. (E) Expression profiles of representative markers for ten distinct cell types. (F) InferCNV heatmap displaying CNVs across cells, with normal samples in the upper panel and tumor samples in the lower panel. Red and blue indicate CNV gains and losses, respectively. [file Image1.tif]

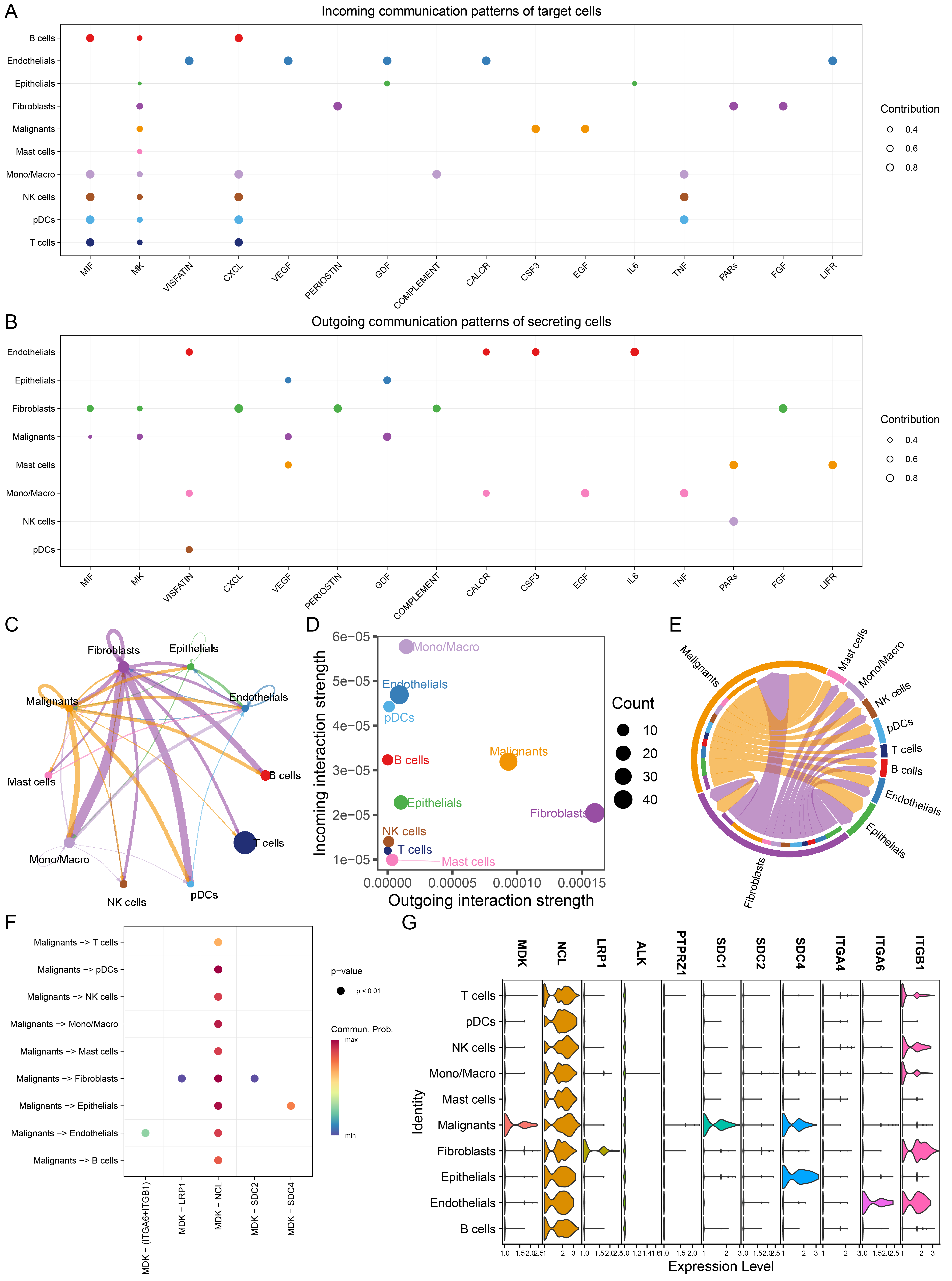

Supplement: Supplementary Figure 2 — Single-Cell communication networks for validation dataset. (A) Incoming communication patterns of target cells, showing pathways to which each cell type responds. (B) Outgoing communication patterns of secreting cells, illustrating the pathways through which cells send signals. (C) Network diagram showing the strength of intercellular communication, with connections between various cell types. (D) Scatter plot comparing outgoing and incoming communication strengths across cell populations, with bubble size indicating the number of interactions. (E) Chord diagram depicting communication via the MK pathway between different cell types. (F) Ligand-receptor interaction probabilities within the MK pathway between malignant and other cell types. Dot size represents significance, and color represents communication probability. (G) Violin plots of MK pathway gene expression levels across cell types, showing gene activity variations. [file Image2.tif]

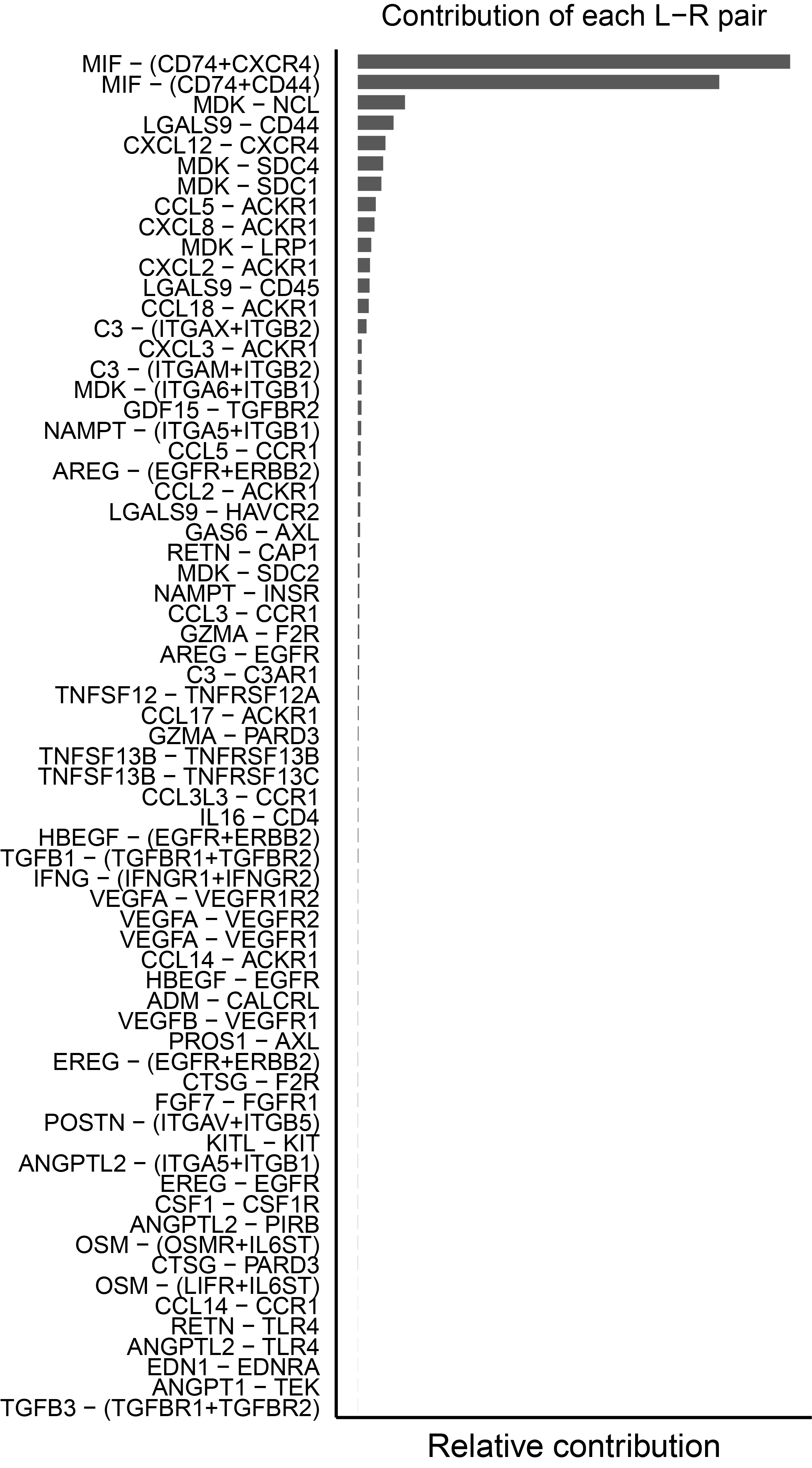

Supplement: Supplementary Figure 3 — Contribution of each ligand-receptor pair for GSE131907 dataset. [file Image3.tif]

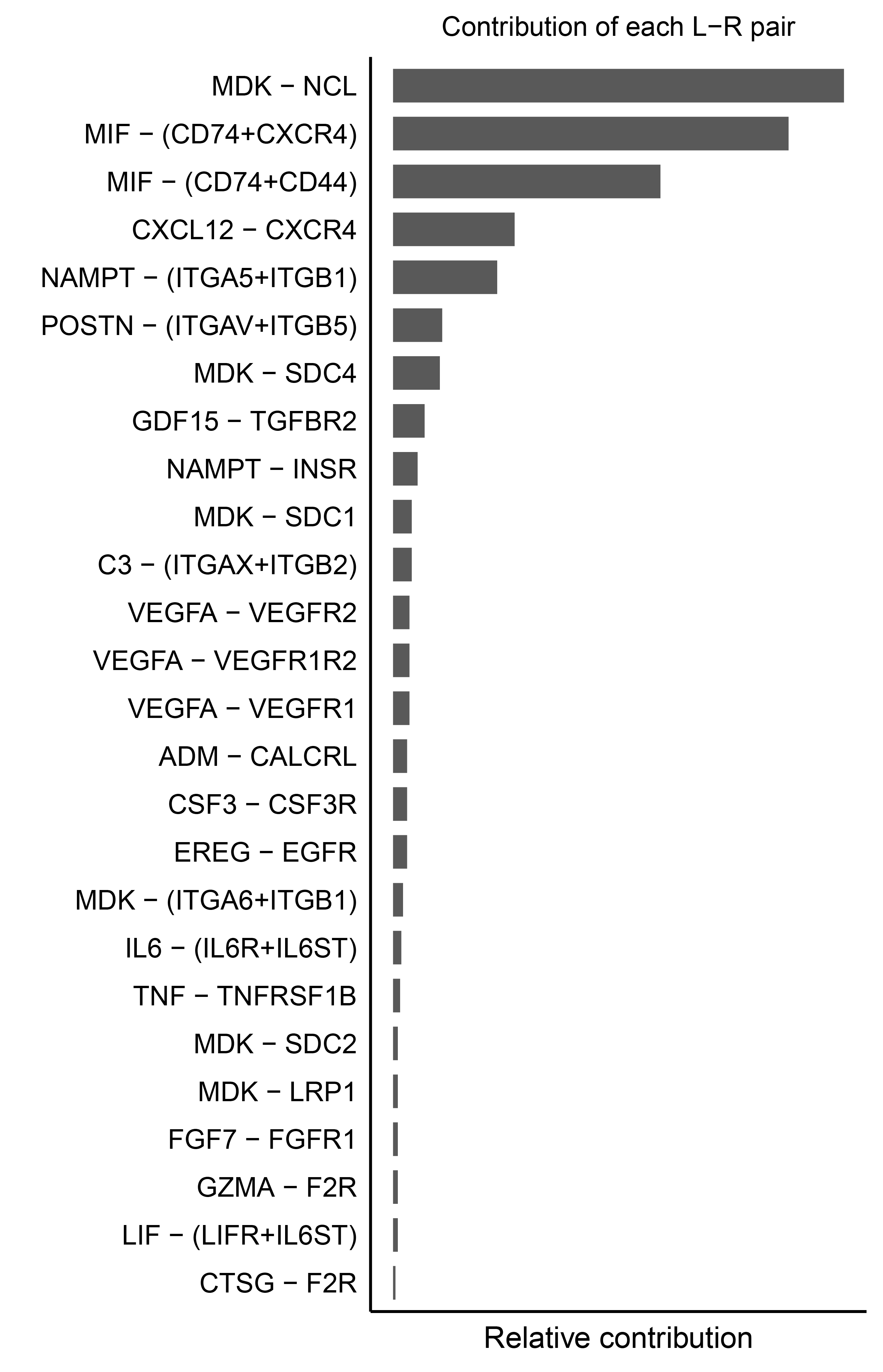

Supplement: Supplementary Figure 4 — Contribution of each ligand-receptor pair for GSE153935 dataset. [file Image4.tif]

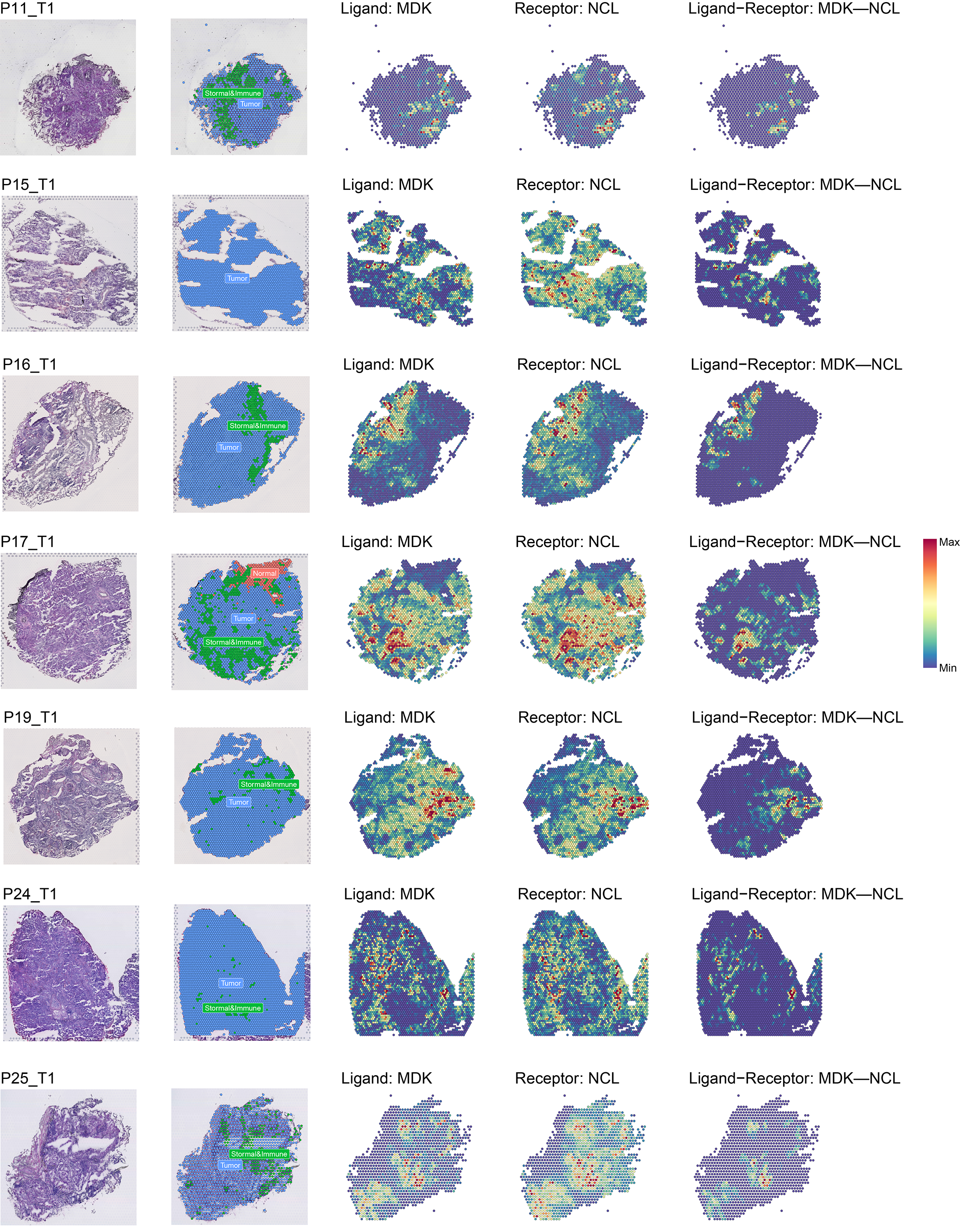

Supplement: Supplementary Figure 5 — The spatial distribution of MDK-NCL receptor-ligand signaling across the niches. [file Image5.tif]

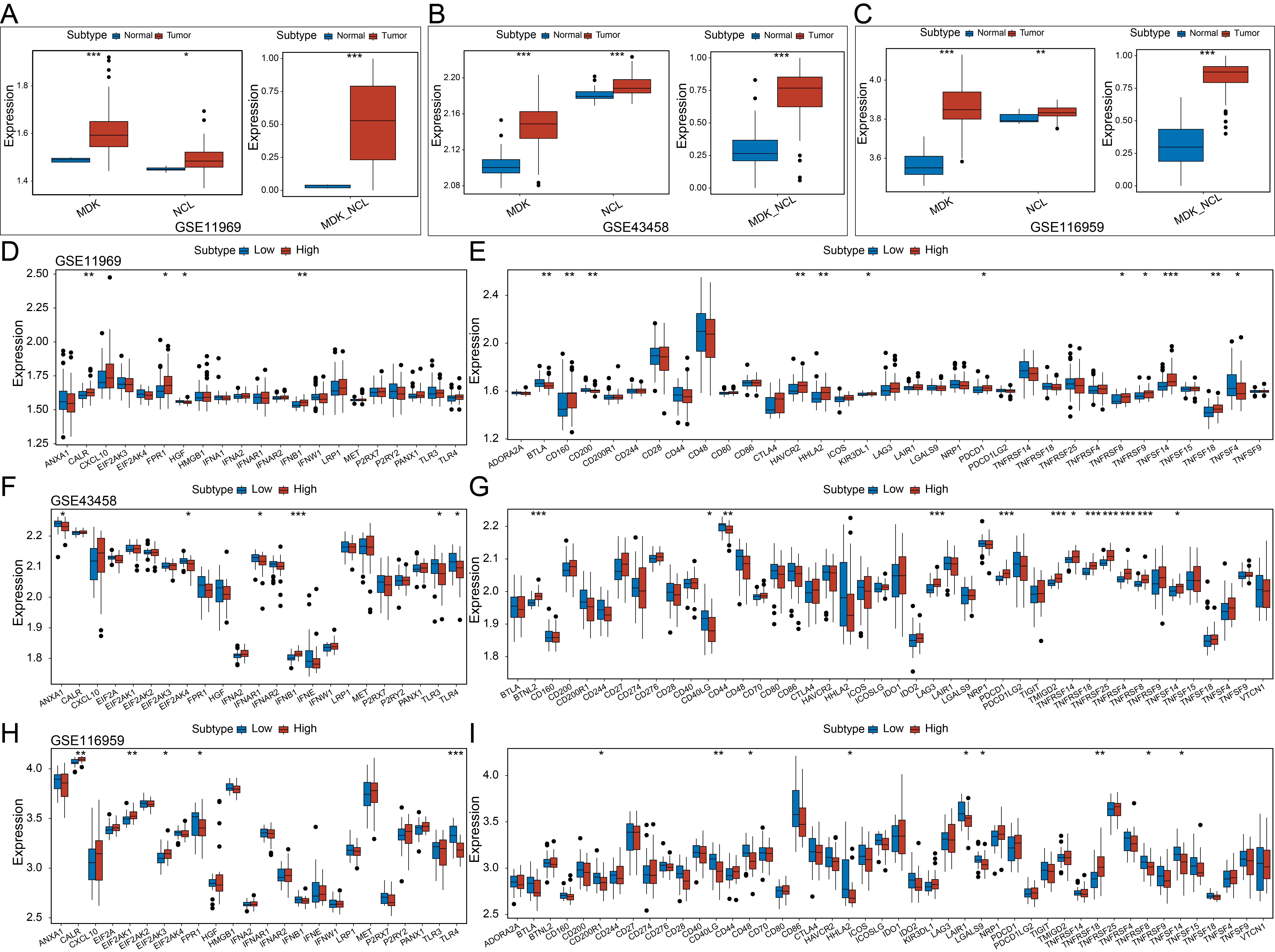

Supplement: Supplementary Figure 6 — MDK-NCL ligand-receptor interaction analysis, spatially mapping MDK ligands, NCL receptors, and their binding regions in validation data. [file Image6.jpeg]
